# Superior plasticity stability and excellent strength in Ti-55531 alloy micropillars via harmony slip in nanoscale α/β phases

**DOI:** 10.1038/s41598-019-41574-7

**Published:** 2019-03-25

**Authors:** Wenjuan Kou, Qiaoyan Sun, Lin Xiao, Jun Sun

**Affiliations:** State Key Laboratory for Mechanical Behavior of Materials, Xi’an Jiaotong University, Xi’an, Shaanxi 710049 P. R. China

## Abstract

Excellent stability of plasticity and high strength are acquired in Ti55531 alloy micropillars via introducing a high density of deformable nanoscale α phase into a β matrix. The yield strength of the pillars is as high as 2.26 GPa irrespective of pillar sizes ranging from 6 to 0.3 μm, which is high enough to activate dislocation slip both in ductile α precipitates and the β matrix. The harmony slip model was proposed to interpret slip transmission between the nanoscale α phase and the divided β matrix, and both α and β accommodate their individual plasticity during compression. This results in an excellent combination of high strength and stable plasticity in Ti55531 alloy micron-to submicron pillars. The results highlight the novel strengthening and toughening mechanisms of nanostructured alloys and a specific type of microstructure that exhibits stable plasticity for nano/microdevices.

## Introduction

It has been well recognized that extremely high strength can be achieved by reducing crystal dimensions to submicron or nanometer scales, the size effect for which “the smaller, the stronger”^1–3^. Two primary mechanisms including the dislocation starvation mechanism^4–7^ and single-arm source mechanism have been proposed to interpret the size effect^8–10^. In these scales, however, plastic instability, such as strain bursts, strain softening or low strain hardening rates, becomes a significant issue due to the avalanche-like dislocation and escape of elements from the crystals, such as Au^4–6^, Ni^1,8^, Cu^11^, Mo^12^, W, Ta, Nb^13^, Al^14^, Mg^15^, Ti^2^. Three-dimensional discrete dislocation dynamics simulations have revealed that strain bursts are statistically characterized by a universal probability distribution whose characteristic parameter is determined by the specimen size^16^. Large strain fluctuations make it difficult to control the resulting shape during plastic formation when the sample dimensions decrease to micrometre and submicron scales^16^. Experimental results have shown that the strain bursts result from the reconstruction of jammed dislocation configurations for pillars greater than 300 nm and from the absence of jammed dislocations for pillars less than 300 nm^17^. Plastic instability deteriorates the service reliability of a mechanical system, and thus hinders the application of crystalline materials with submicron or nanometer dimensions in micro-electro mechanical devices.

To mitigate plastic deformation instability, two strategies have been proposed to restore the mean-field condition of dislocation interactions by introducing fewer scales of defects to trap dislocations^18–20^, including grain boundaries^21^ and precipitates^22,23^. Embedding dispersed precipitates into the matrix is widely employed to suppress intermittent and stochastic strain bursts^22–24^. Girault *et al*.^23^ proposed that the spacing of the internal precipitates controlled deformation behaviour. Plastic instability was eliminated by introducing Ni-based oxide-dispersion in the Inconel MA6000 micropillars greater than 1 μm. Gu *et al*.^25^ discovered that precipitates in 2025-Al alloy micropillars resisted and slowed dislocation motion, resulting in enhanced work hardening and weak size dependence of pillar strength, but strain bursts were still obvious when the pillar size was less than 2 μm. Chen *et al*.^22^ discovered that a dense uniform dispersion of silicon carbide nanoparticles in magnesium can be achieved through a nanoparticle self-stabilization mechanism in molten metal, but the strain-stress curves were not smooth.

Strengthening particles such as intermetallic compounds, oxide particles, and carbides usually have an extremely high strength^21,23,26–28^. For example, the strength of SiC particles is nearly 3 GPa^29^. Mg_2_Zn small-scale samples with nanoscale SiC particles yield strengths of approximately 410 MPa^22^. This indicates that the strength of silicon carbide is much higher than that of Mg_2_Zn-SiC micropillars. The precipitate strengthening mechanism consists of the Orowan by-pass mechanism and dislocation cut through mechanism^21,23,26–28^. For some shearable obstacles, once the dislocations overcome the barrier of strengthening particles, an easy path for dislocation movement is established in the materials. Collective dislocation moving out of the sample may take place. This is a predominant mechanism of strain burst in pillars that contain strengthening particles.

In this study, we demonstrated that by introducing a deformable nanoscale α phase, plastic instabilities can be dramatically suppressed in micron- to submicron-scale Ti55531 pillars. The phase interface between the α precipitates and the β matrix plays a role in storing substantial dislocations and initiating dislocation sources to mediate plasticity. Strength and plasticity stability can be tailored between the second phase and the matrix. The goal of this paper is to elucidate the strengthening and toughening mechanism of nanostructured components and nano-scales of alloys, as well as to pave a way for their large-scale industrial applications in nano/microdevices.

## Results

**Figure 1:**
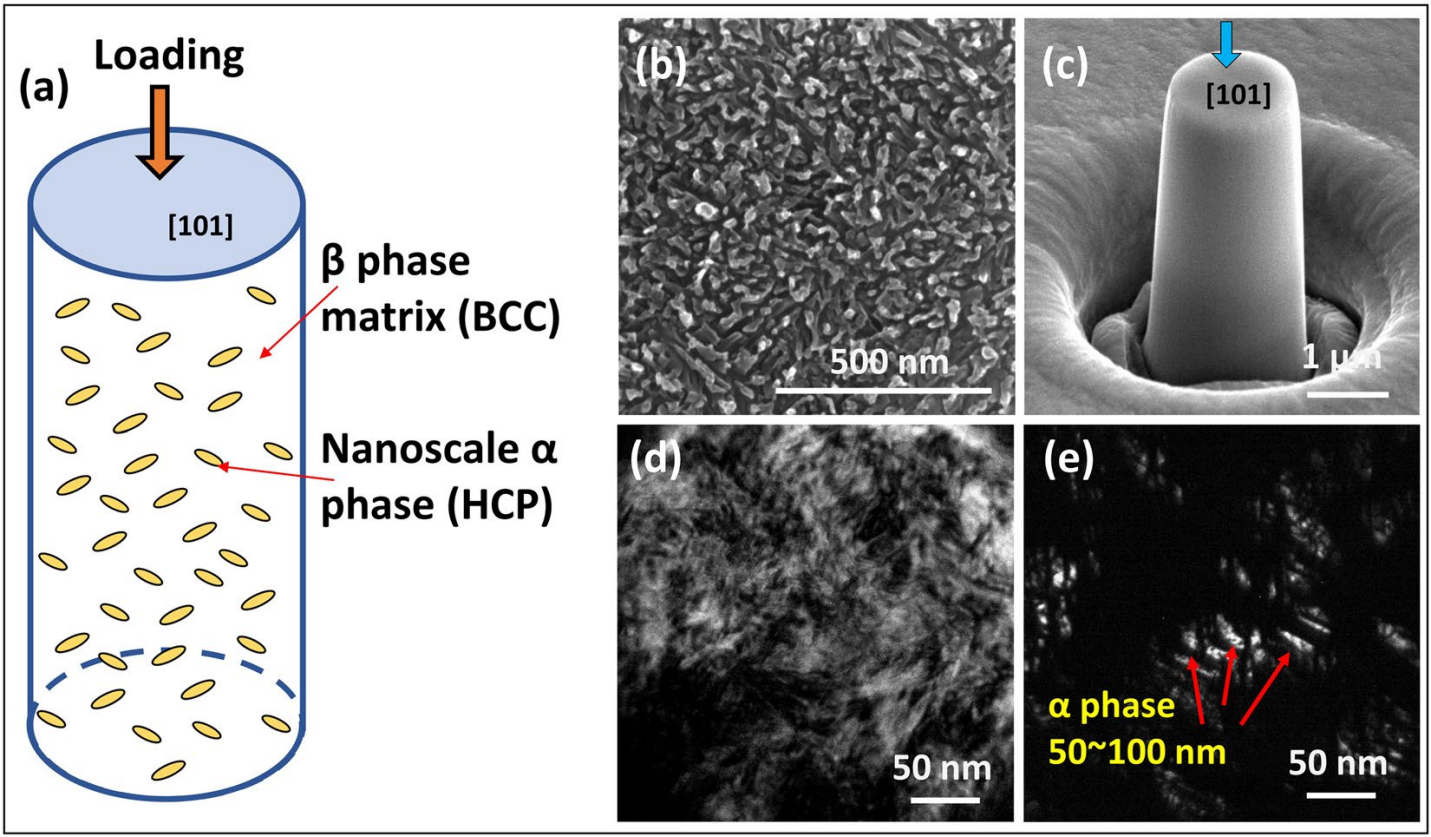
Microstructure and schematic illustration of loading mode in Ti55531 alloy pillars before deformation. (**a**) Schematic illustration of Ti55531 alloy pillars containing nanoscale α phase in β matrix subjected to compressive loading. (**b**) SEM morphology. (**c**) The fabricated micropillar and loading direction. (**d**) Bright-field image of substructure. (**e**) Dark-field image of substructure.

Figure 1(a) is a schematic diagram of a micropillar containing a nanoscale α phase and its loading direction. Figure 1(b,e) show the microstructure of Ti55531 containing a high density of uniformly distributed α phases in the β matrix before deformation. The micropillar orientation was determined to be [101] in the β matrix. The aspect ratio (height to diameter ratio) of pillars is 2.3–2.6, as shown in Fig. 1(c). Figure 1(d,e) show the bright and dark images of the undeformed microstructure. The electron diffraction pattern along the <111> β zone axis has a bright additional reflection at the 1/2{1 $\bar{1}$0} position. This α phase was formed during ageing. The morphology of the α phase is clearly exhibited in the dark-field image (Fig. 1(e)). The size of the nanoscale α phase is approximately 50–100 nm. The experimental details are supplied in the methods section.

**Figure 2:**
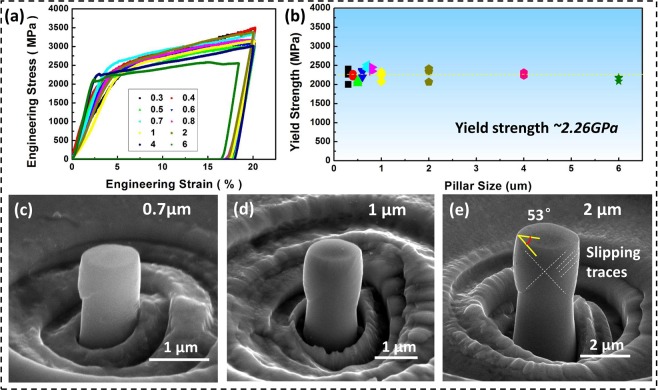
Compressive behaviours of Ti55531 micropillars. (**a**) Engineering stress-strain curves of micropillars of different sizes. (**b**) Yield strength (at 1% strain) versus pillar diameter curves. The average yield strength is approximately 2.26 GPa. (**c**–**e**) SEM morphology of deformed micropillars with different diameters.

Figure 2(a) depicts engineering stress-strain curves of micropillars ranging from 6 μm to 0.3 μm in diameter. The yield strength, which is defined as the flow stress of the micropillar at 1% engineering strain, remains at approximately 2.26 GPa, regardless of pillar sizes, as shown in Fig. 2(b). The results indicate that Ti55531 pillars do not follow the size effect of “smaller is stronger” at submicron scales. After close examination, the stress-strain curves show continuous and smooth stable plastic deformation behaviour, which is very similar to the bulk samples. In other words, strain burst does not happen during the plastic deformation of Ti55531 micropillars. Ti55531 pillars with a nanoscale α phase exhibit an excellent combination of high strength and stable plasticity.

**Figure 3:**
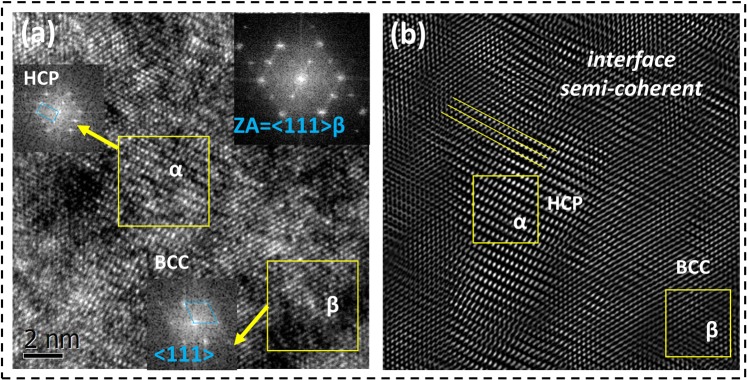
HRTEM images of Ti55531 micropillars before deformation. (**a**) HRTEM image of the β/α interface. (**b**) Inverse Fourier transform of Fig. 3(a).

Figure 2(c–e) show scanning electron micrographs of the deformed Ti55531 pillars. Very tiny slip traces were observed on the surfaces of pillars, in contrast to the significant slip steps observed in other pure metallic pillars^1–6^. When the compression strain reaches 20%, the pillars still maintain plastic stability. The angle between the top surface and the slip trace was measured to be approximately 53°. The crystallographic slip system is determined to be {112} <111> slip, which possesses a Schmid factor of 0.471 when the compression is along the [101] orientation. Figure 3(a,b) show high-resolution transmission electron microscope (HRTEM) images of the undeformed micropillars. The electron diffraction pattern is along the <111> β zone axis. The β matrix has a common β-{110} pole with the α-{0001} pole. The α precipitates and the β matrix have the following Burgers orientation relationship: {110}_β_//{0001}_α_ and <1 $\bar{1}$1>_β_//<11$\bar{2}$0>_α_^30–32^. A semi-coherent interface between the α precipitates and β matrix is exhibited, as shown in Fig. 3(b), which is the inverse Fourier transform of Fig. 3(a).

**Figure 4:**
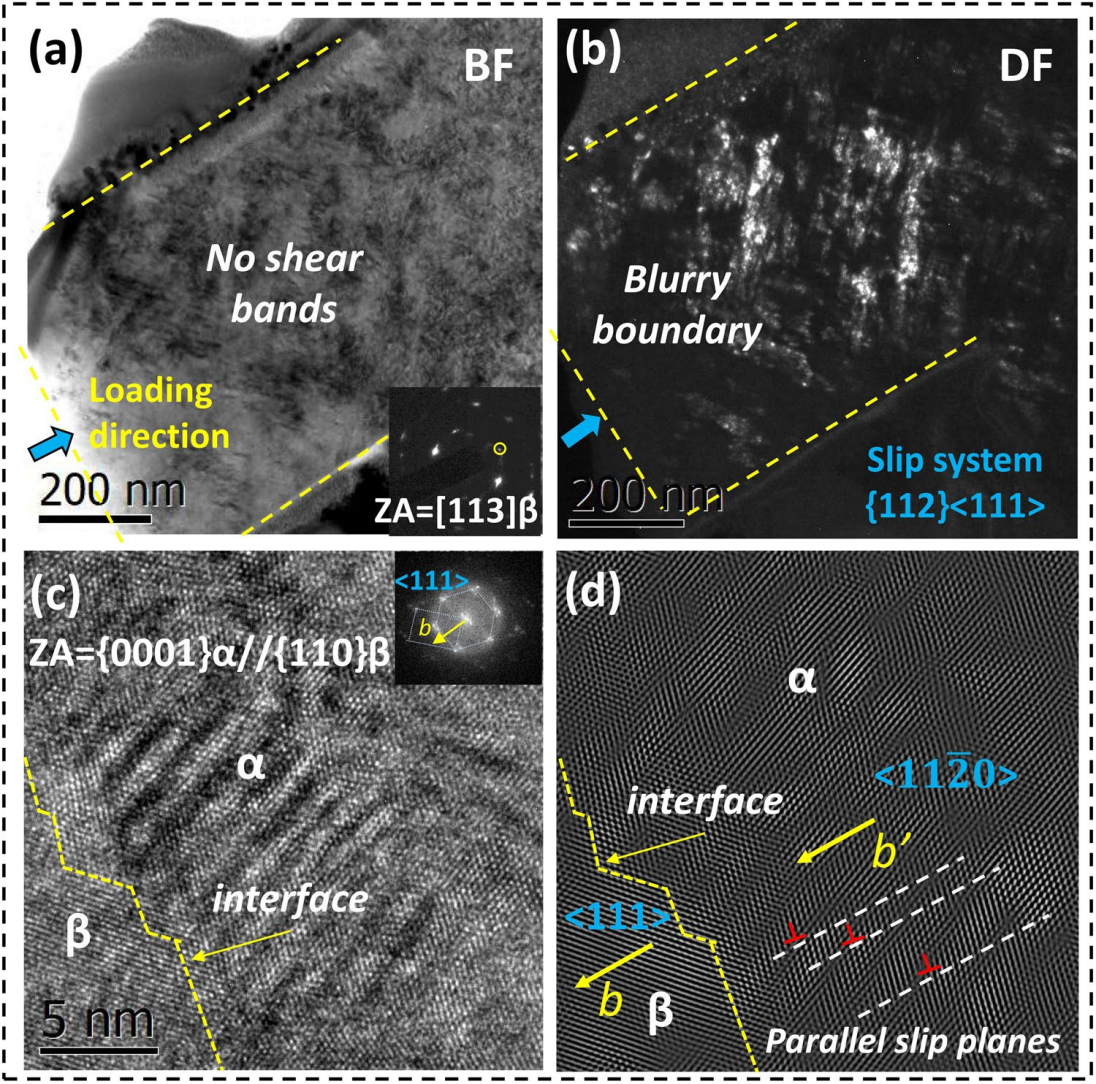
TEM microstructures of deformed Ti55531 micropillars. (**a**) Bright-field image of a deformed pillar. (**b**) Dark-field image of a deformed pillar. (**c**) HRTEM image of the β/α interface and deformed α phase. (**d**) Inverse Fourier transform of (**c**).

Figure 4(a,b) show the bright-field and dark-field images of the deformed micropillars, respectively. The electron diffraction pattern is along the <113> β zone axis. No shear bands can be distinguished. Figure 4(c) shows a HRTEM image of the β/α interface. The electron diffraction pattern is along the <110> β zone axis. The same Burgers orientation relationship is observed between α precipitates and the β matrix in the deformed pillars: {110}_β_//{0001}_α_ and <1 $\bar{1}$1>_β_//<11$\bar{2}$0>_α_^30–32^. Compared to the undeformed micropillar in Fig. 3(a), more dislocations were observed in the deformed pillars (Fig. 4(c)). By inverse Fourier transform, some dislocations are located near the β/α interface, as shown in Fig. 4(d). By comparing the microstructures before and after compression with HRTEM, dislocations were observed both in the nanoscale α phase and in the β matrix. This result indicates that the α phase and β matrix simultaneously undertake the plasticity. Dislocations were observed inside the α precipitates and the Burgers vector $\mathop{{\bf{b}}\text{'}}\limits^{\rightharpoonup }$ is along <11$\bar{2}$0 > direction, as shown in Fig. 4(d). According to the Burgers orientation relationship, <1 $\bar{1}$1>_β_//<11$\bar{2}$0>_α_, and the $\mathop{{\bf{b}}}\limits^{{\boldsymbol{\rightharpoonup }}}$ of the β matrix is parallel to <111> slip direction. At the same time, the dislocations slip in several parallel planes inside nanoscale α phase, as indicated by the dotted line in Fig. 4(d).

## Discussion

### Excellent plastic stability in Ti55531 pillars

Micron- to nanoscale acicular α precipitates usually play an important role in strengthening β-titanium alloys. As a hexagonal closed-packed (hcp) structure, the frequent slip systems in the α phase include {10$\bar{1}$0} prismatic slip, {0001} basal slip and {10$\bar{1}$1} and {10$\bar{2}$2} pyramidal slips. The critical resolved shear stress (CRSS) of pyramidal and basal slips is higher than that of the prismatic slip^33,34^, as listed in Table 1. However, in the precipitates strengthened with β-titanium alloys, the strengthening effect results from submicron- to nano-scales of the α phase in β matrix. Since we know that the strength of the α phase is dependent on its size, it exhibits the “smaller is stronger” trend^35^. The compression of micropillars is an available and relatively viable technique to acquire the yield strength of nanoscale α phase itself. The external constraints do not have a significant effect on the intrinsic strength of α phase^36,37^. The strength of α phase itself is the dominant factor. So the strength of α precipitates can be calculated with the following power Equation (1)

1 $${{\rm{\sigma }}}_{{\rm{\alpha }}}={{\rm{k}}}_{{\rm{\alpha }}}\ast {{\rm{d}}}^{{\rm{n1}}}$$

where the power exponent n_1_ is −0.44^35^ and the constant k_α_ is 620, according to the experimental data for Ti-6Al micron-scale cantilevers^38^. The average size of the α phase is estimated to be 60 nm according to Fig. 1(b,c). The yield strength of the α phase (σ_α_) is calculated to be approximately 2.14 GPa, which means that the nanoscale α phase is yielded only when the applied stress reaches 2.14 GPa. However, the yield strength of bulk Ti55531 alloy is reported to be 1.1–1.3 GPa^39,40^ because there is grain boundary α and primary α, of which both sizes are in microns and cause the bulk alloy yield at lower stress, far less than the strength of the nanoscale α phase (approximately 2.14 GPa). In bulk β phase Ti alloys with high strength, the α phase is considered to be undeformable in the β matrix^41^. However, the yield strength of Ti55531 pillars is approximately 2.26 GPa in compression, which is the same order of magnitude as the strength of the nanoscale α phase. As such, the nanoscale α phase becomes deformable during the compression of micron- to submicron-scale Ti55531 alloy pillars.

**Table 1:** CRSS for different slip systems in nanoscale α phase^33,34^.

Slip system	CRSS(MPa)
prism slip	350
basal slip	450
pyramidal slip	850

For the Ti55531 alloy micropillars within one β grain, the microstructure is composed of nanoscale α precipitates and a β matrix. Due to the dense and homogeneous α precipitates in the β matrix, as shown in Fig. 1(b), a β matrix divided by α precipitates is similar to an interconnected net. Dislocation slip is probably limited in the divided β matrix with a submicron size due to the large number of α/β interfaces. There have been no strength data produced for the β phase at the submicron scale for the Ti55531 alloy until now. Small pillars are used to measure the strength of the β phase Ti55531 alloy, and the strength of the β phase pillars with the same order of magnitude of divided β matrix (details in Supplementary Materials) can be taken as the strength of the β matrix for the Ti55531 pillars. The strength is calculated with the following equation:

2 $${{\rm{\sigma }}}_{{\rm{\beta }}}={{\rm{k}}}_{{\rm{\beta }}}\ast {{\rm{d}}}^{{\rm{n2}}}$$

where the exponent of the power law n_2_ is −0.33 for the β phase and the constant k_β_ is 1167.7, which are regressed with the strength of the β phase micropillars. The fitting detail is shown in Supplementary Fig. S1. The divided regions of the β matrix range from 50–150 nm based on examination and statistical data from the scanning electron microscopy (SEM) and transmission electron microscopy (TEM) images, as shown in Fig. 1(b,d). The σ_β_ is calculated to be 3.1-2.1 GPa according to Equation (2), depending on the size of the divided β matrix. Because the applied stress of pillars in compression increases to greater than 2.26 GPa after yielding, both the α phase and β phase are considered to accommodate plastic deformation.

Based on the above results, the yield strength of the micropillars (2.26 GPa) is the same order of magnitude as those of the nanoscale α phase and the β matrix. Therefore, both the α phase and β phase participate in plastic deformation during the compression of Ti-55531 alloy pillars. The elasticity modulus of the α phase (HCP) is higher than that of the β phase (BCC) in titanium alloys. The α phase undertakes higher stress than the β phase for the same strain of pillars. As a result, it is reasonable to assume that slip could first occur in the α phase and then go into the β phase. In fact, slip in the α phase is first observed in the deformation of bulk α/β titanium alloy^42^. During the compression of pillars, when the applied stress reaches the yield stress, dislocation slip occurs in some α with favoured orientations. The α/β interfaces are not hard barriers against slip because the Burgers orientation relationship between the α phase and the β phase. The dislocation Burgers vector is 0.295 nm in the HCP α phase and is 0.289 nm in the BCC β matrix. In addition, the P-N Stress in HCP is less than the BCC crystalline structure^42^. An increase in stress is necessary for slip transmitting to the α/β interface^43,44^. Dislocations pile-up lead to stress concentration, which causes an increase in flow stress and strain hardening such as in a bulk alloy. The increase in flow stress can activate slip in the α phase with a lower Schmid factor as well, and this results in a further increase in flow stress. Therefore, the α interface plays an important role in maintaining a continuous increase in flow stress and strain hardening for Ti55531 alloy pillars during deformation.

**Figure 5:**
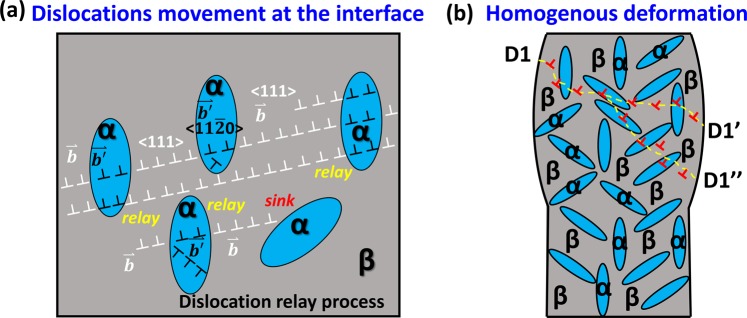
Schematic diagram of the “slip relay” model between dislocations and interfaces. (**a**) Dislocation movement between the interfaces. <111> dislocation $\mathop{{\rm{b}}}\limits^{{\rm{\rightharpoonup }}}$ in β matrix is parallel to <11$\bar{2}$0> dislocation $\mathop{{\rm{b}}\text{'}}\limits^{\rightharpoonup }$ in the α phase. Both the β matrix and α phase have their own slip systems. (**b**) Plastic stability by a zig-zag slip path.

This harmony slip mode in the Ti55531 alloy pillars is similar to “slip relay” behaviour, which is schematically shown in Fig. 5. The α variants with different orientations are distributed in the β matrix. First, slip occurs in some α precipitates with a high Schmid factor, and then slip meets interface barriers and results in dislocations pile-up and stress concentration at interfaces. Then, local stress concentration leads to an increase in flow stress and slip activation in the neighbouring β phase, as well as activating slip in the α precipitates with lower Schmid factors. Slip must meet interface barriers frequently due to the submicron-scale mean free path of dislocation in the β matrix, following the sequence “slip, dislocation pile-up, stress concentration, slip at higher stress”, which results in a continuous increase in flow stress. The main cause for the slip mode is the ductile α and β phases, both with comparable yield strengths and both of which possess enough slip systems that are activated at critical stress. In difference with the traditional shearable particles, the deformable nanoscale α phase has the hardening capability of plastic deformation without strain concentration inside shear band. As a result, the dislocations slip in several parallel planes inside nanoscale α phase, as shown in Fig. 5(a). The dislocations can be accumulated and stored inside α phase and result in work hardening and stable plastic deformation. During compression of Ti55531 pillars, slip proceeds from the α to β phase and transmits interfaces with dislocation pile-up and local stress concentration at interfaces, and this gradual slip mode causes stable plasticity in micropillars. Such a slip mode is considered to be harmony slip, which means that both the ductile α and β phases accommodate plastic strain via slip without stiff barriers that cease dislocation movement. Generally, slip is the most frequent plastic deformation in alloys. The interfaces and grain boundaries are barriers against slip and have been used to strengthen bulk alloys in engineering applications; however, trans-interface slips have been observed and have attracted more attention in recent years^42,45–48^. In Ti55531 micropillars with the nanoscale α phase in β matrix, the two phases maintain the Burgers orientation relationship {110}_β_//{0001}_α_ and <1 $\bar{1}$1>_β_//<11$\bar{2}$0>_α_. It is worth noting that the plane and direction in Burgers orientation relationship correspond to the slip plane and direction in the α and β phases, respectively. This indicates that some slip systems are well-aligned and slip can transfer through the interfaces and cause slip to transfer from one phase to another. It is reported that not only slip but also twinning, is possible to transfer through interfaces when the geometric alignment of slip or twinning systems and an interface with fewer defects exists in the interface^49^.

### High strength of the Ti55531 micropillars without size effect

Size dependence of strength is a common feature of metallic micropillars and exhibits a power law for the micron- to submicron scale. A parameter, an exponent of the power law, is suggested to characterize the size effect, and it is always negative to accommodate for the understanding “smaller is stronger”. The exponent of the power law is an approximate constant of −0.6 for face-centred cubic (FCC) metals^35^. However, for body-centred cubic (BCC) metals, it depends on the Peierls barrier. From low to high Peierls barriers, the exponents are −0.21 and −0.93 for W and Nb^13,50^, respectively. For HCP metals, the exponent of the power law is dependent on deformation modes^35,51^. It is found that the exponent of the power law depends on the multiplication and interaction of dislocation slip in metallic micron pillars. The Peierls barrier energy is related to the amount of hard junctions^24,52^. The investigation shows that more mobile dislocations interact to form hard junctions more in BCC metals compared to FCC metals, and the latter exhibit increased size dependence on strength than the former at the microscale. However, an interface and second phase are introduced into micropillars to modify the size effect on strength. When the pillars are composed of secondary particles in the matrix, serious interactions between the dislocation and secondary particles weaken the size effect. For some alloys with secondary particles, the strengthening effect that results from internal hardening is superior to the external size effect, with a decreasing absolute value of the exponent. For Ni-based superalloy MA6000, the exponent of the power law decreases to −0.045^23^. However, the size effect will be displayed when the sample size decreases to less than 1000 nm. R. Gu^25^ reported that the exponent decreases to −0.07 in duralumin micropillars via a dislocation by-pass mechanism. The Al7075-SC micropillars also show an obvious size effect and heterogeneous plastic deformation^21^. For some specific alloys, the grain boundary or phase interface is introduced as a dislocation source in the Ni micropillars and results in a “smaller is weaker” effect with an exponent of 0.15^52^.

**Figure 6:**
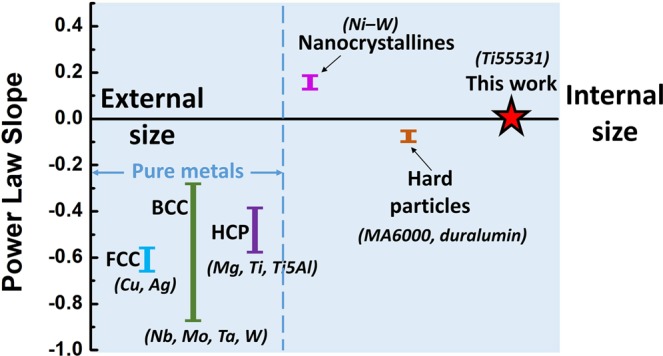
Exponent of power law – external/internal size relationship of various alloys and materials in microscales.

However, our results show that the exponent of the power law is close to zero for the Ti55531 alloy, but the micropillars still exhibit stable plasticity even when the size decreases to 300 nm. The exponents of the pillars reported in the literature are summarized in Fig. 6. Size-dependent mechanisms such as dislocation starvation or source exhaustion are not valid in precipitates strengthened with a Ti55531 alloy.

The internal size of the nanoscale α phase is believed to dominate the strength of micropillars, instead of the external size of the pillar. The high density of internal obstacles (α phase) leads to a submicroscale mean free path of dislocation motivation inside the β matrix and dominates the strength of the pillar by replacing the external pillar size. Dislocations can hardly transmit the β/α interface directly due to the differences in crystal structure and lattice constant. As a result, the mean free path of dislocations depends on nanoscale α phases^25,53^ and governs the strength of micropillars^54^. The β matrix is separated by the α phase and dislocation movement is limited in small regions, as shown in Fig. 5(b). Since the slip system is probably first activated in the nanoscale α phase, the high yield strength of pillars is attributed to the interaction between dislocations and a large number of α/β phase interfaces. Based on the Taylor model, if the second phase is introduced in the matrix, the flow stress is described as follows^55–57^:

3 $${\rm{\tau }}={{\rm{\tau }}}_{0}+{\rm{C}}{\rm{\mu }}{({\rm{br}}/{\rm{\lambda }})}^{1/2}$$

where C is a constant, μ is the shear modulus, b is the Burgers vector, r is the radius of second particles, and λ is the dislocation slip distance, which is dependent on the microstructure^55–57^. Since the shear modulus μ and Burgers vector b have constant values, these values and constant C can be replaced by a coefficient k. (λ/r) is defined as L, which is dependent on the size of nanoscale α in the β matrix. Equation (3) can be simplified as follows:

4 $${\rm{\sigma }}={{\rm{\sigma }}}_{0}+{{\rm{kL}}}^{-1/2}$$

where k is a constant and L is dependent on the size of nanoscale α. σ_0_ is the critical flow stress of the matrix, which represents the resistance to dislocation motion by other factors, except the nanoscale α phase. Here, we define the σ_0_ as the critical flow stress of Ti55531 without an α phase. The critical flow stress of the matrix, σ_0_, is approximately 0.9 GPa, which is acquired by the power law relationship of the β matrix, as shown in Supplementary Fig. S1. The constant k is 1.01, acquired by the experimental results of 650 °C ageing Ti55531 micropillars, and the fitting detail is shown in Supplementary Fig. S3. As such, the strength of 450 °C ageing Ti55531 micropillars σ is calculated to be 1.9 GPa, which is close to the experimental result of 2.26 GPa. The results show that microstructure with a nanoscale α phase in β matrix exhibits both high strength and stable plasticity (without strain bursts) due to the comparable strength of α and β, and both phases participate in accommodating plastic strain during deformation. This feature has potential for micron- to nanoscale devices and components with strict size requirements.

## Conclusion

A combination of superior plastic stability and high strength have been acquired in Ti55531 microscale pillars by introducing a high density of nanoscale α phases. A harmony slip model in which both the ductile phase accommodates plasticity with slip relay during deformation was proposed to interpret the stable plasticity and without strain bursts. The yield strength of micropillars reaches approximately 2.26 GPa regardless of pillar size, for pillars from 6 to 0.3 μm. The strength of Ti55531 alloy micropillars is predominated by the dense and homogeneous nanoscale α phase and divided β matrix, instead of external pillar size. A kind of nanostructure is designed to exhibit mechanical behaviours similar to bulk alloys. In addition, the results not only shed light on novel strengthening and toughening mechanisms in nanostructured alloys but also pave a way for the potential applications in MEMs devices with stable size requirement.

## Methods

Bulk polycrystalline Ti-5wt%Al-5wt%Mo-5wt%V-3wt%Cr-1wt%Zr (Ti-55531) was chosen in this work. A series of heat treatments were conducted to obtain a high density of uniformly distributed α phases. First, polycrystalline Ti-55531 bulk samples that were 4 mm × 4 mm in cross-section and 7 mm in length were solution heat-treated at 950 °C for 24 h in a high vacuum furnace followed by water quenching. The achieved β grain size was in the range of 0.5–1 mm. Further isothermal vacuum annealing was performed at 300 °C for 20 h to form a large number of athermal ω precipitates, which can become the nucleus of the stable α phase nucleation^58–60^. The annealing temperature was further increased to 450 °C for 15 h to dissolve the remaining ω and promote the growth of nanoscale α precipitates by clustering different α variants into self-accommodating morphologies^59^.

The square bulk was mechanically and electrochemically polished before fabricating it into pillars. The grain orientation was determined via electron backscattered diffraction (EBSD). A group of submicrons of pillars were fabricated using the FEI Helios Dual Beam Focus Ion Beam (FIB) microscope operated at an ion beam voltage of 30 kV with a variety of currents. The pillar diameter was 6 μm to 0.3 μm. Three to five samples were prepared for each size. Micro-compression testing was performed in the Hysitron TI950 nanoindentation with a flat tip, at a nominal axial strain rate of 1 × 10^−3^s^−1^. The post-compression morphology of the pillars was examined with SEM and TEM. All TEM specimens were lifted out using a nanomanipulator (AutoProbe 200, Omniprobe, Inc.) and were transferred onto Cu TEM grids. The pillar samples were then thinned to the electron-transparent thickness (approximately 100 nm) by using the focused ion beam (FIB).

## Supplementary information

supplementary
