# Supplementary material for: Superior plasticity stability and excellent strength in Ti-55531 alloy micropillars via harmony slip in nanoscale α/β phases
Source: Sci Rep. 2019 Mar 25;9:5075. doi: 10.1038/s41598-019-41574-7 (PMC6433921; doi:10.1038/s41598-019-41574-7)
Supplement: Supplementary file 1 — supplementary [file 41598_2019_41574_MOESM1_ESM.docx]

**Supplementary Information**

**Superior plasticity stability and excellent strength in Ti-55531 alloy micropillars via harmony slip in nanoscale α/β phases**

Wenjuan Kou, Qiaoyan Sun*, Lin Xiao*, Jun Sun

State Key Laboratory for Mechanical Behavior of Materials, Xi’an Jiaotong University, Xi’an, Shaanxi 710049, P. R. China

* Corresponding authors.

E-mail address: qysun@mail.xjtu.edu.cn (Qiaoyan Sun), lxiao@mail.xjtu.edu.cn (Lin Xiao).

1. **Regression of power law exponent *n* and constant *σ_0_* in pure β-Ti-55531 micropillars.**

Ti-55531 bulk was solution heat-treated at 950℃ for 24 h in a vacuum environment followed by water quench to obtain the pure β-Ti55531. A series of β-Ti55531 pillars with different diameters were fabricated and compressed to establish the relationship between the yield stress and pillar diameter. The yield strength of pure β-Ti55531 micropillars as a function of pillar diameter is shown in Figure S1. They obey by a power law: σ_β_ = k_β_$*D^{-n}$. The constant k_β_ is 1167.7 and power law exponent n is 0.33. Increasing the pillar size, the σ_β_ approaches a constant of σ_0_, which is equal to about 0.9 GPa regardless of the pillar size.


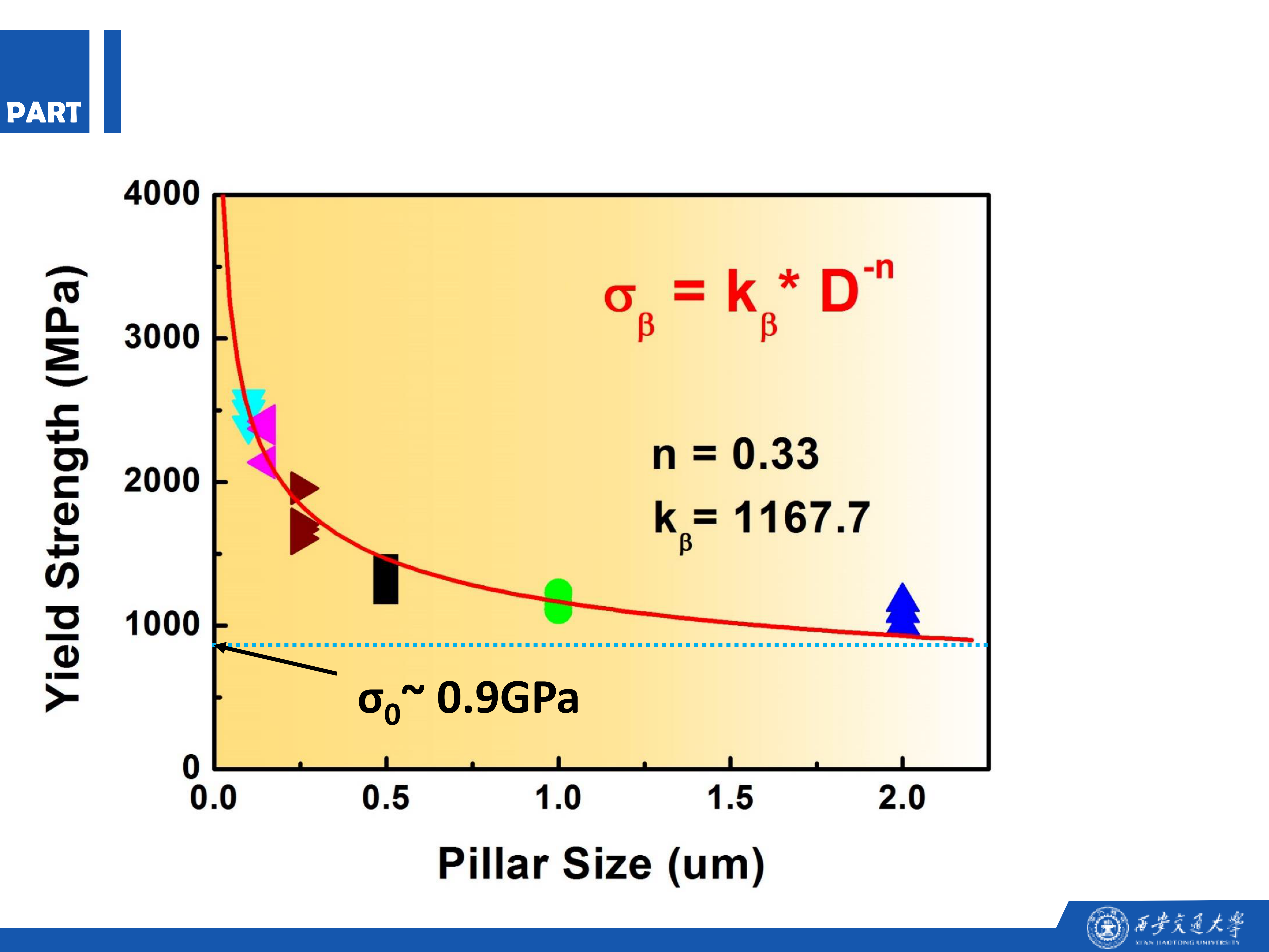


**Supplementary Figure S1. The yield strength of pure β-Ti55531 matrix.** The strength can be regressed with a power law: σ_β_ = k_β_$*D^{-n}$, constant k_β_ is 1167.7 and power exponent *n* is 0.33. σ_0_ is about 0.9 GPa, regardless of the sample sizes.

1. **Regression of constant *k* in the aged Ti55531 micropillars.**

Ti55531 bulk was solution heat-treated at 950℃ for 24 h. The further isothermal annealing was conducted at 300℃ for 20h followed by second annealing at 650℃ for 15h. High density of α precipitates were introduced in the β matrix, as shown in Figure S2. The morphology of α precipitates is short rod-like with 100nm in average width and 800nm in average length. A group of aged Ti55531 pillars containing high density of α precipitates in β matrix were fabricated and compressed. The yield strength of the aged Ti55531 micropillars with different sizes was measured to be about a constant of 1.5 GPa, as shown in Figure S3. No size effect is displayed. The relationship between the parameter L and the yield strength (σ) is described as: σ=σ_0_+kL^-1/2^. The constant σ_0_ and the parameter L were experimentally measured. The constant k is calculated to be 1.01.


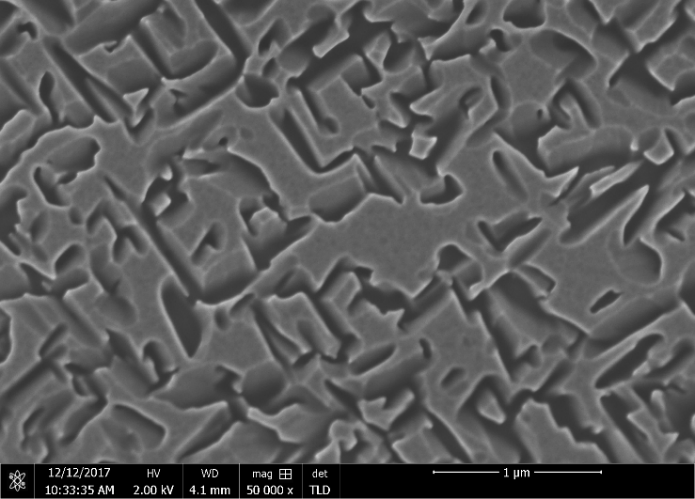


**Supplementary Figure S2. The SEM morphology of α phase in the 650℃ aged Ti55531 samples.**


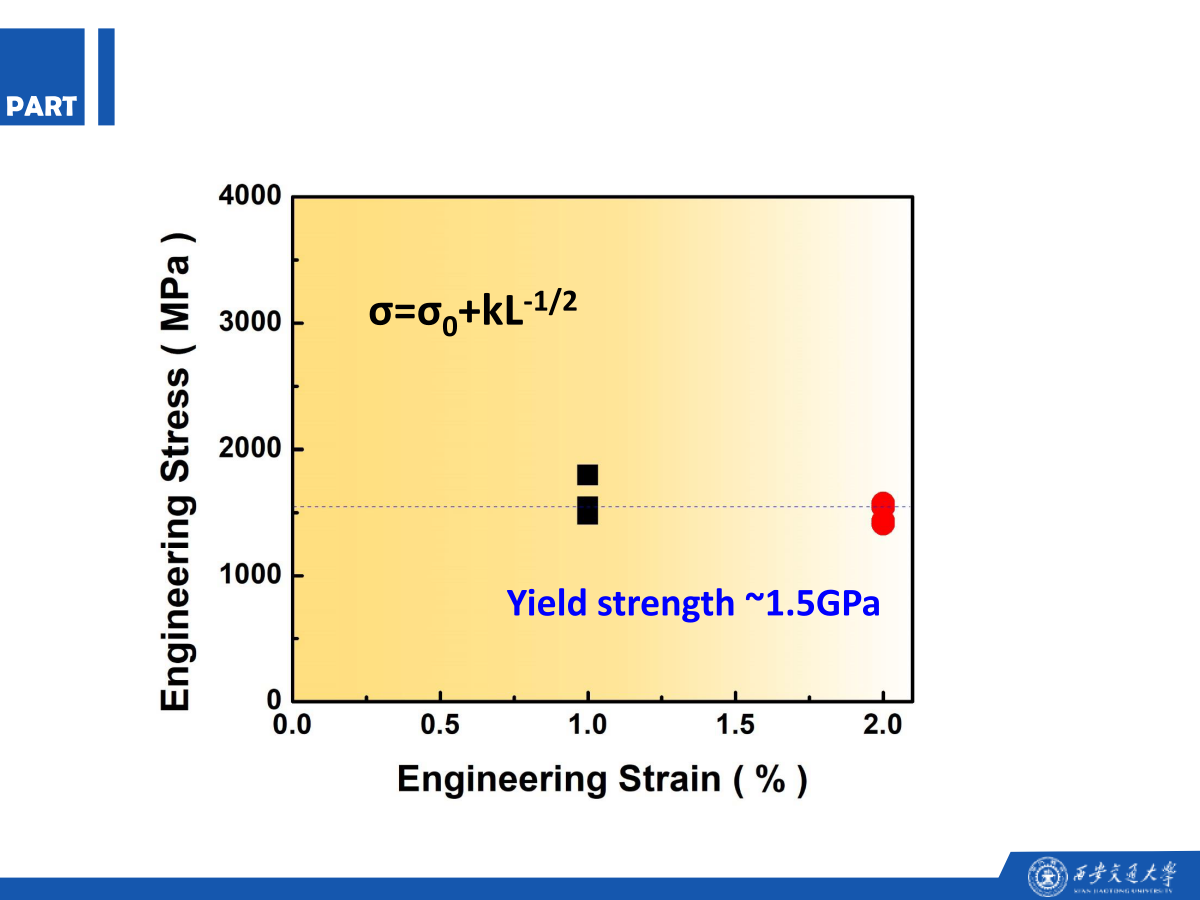


**Supplementary Figure S3. The yield strength of Ti55531 micropillars aged at 650℃.**
